# Image-Based Quantification of Gold Nanoparticle Uptake and Localization in 3D Tumor Models to Inform Radiosensitization Schedule

**DOI:** 10.3390/pharmaceutics14030667

**Published:** 2022-03-18

**Authors:** Ljubica Z. Petrovic, Michael Oumano, Justin Hanlon, Mark Arnoldussen, Igor Koruga, Sayeda Yasmin-Karim, Wilfred Ngwa, Jonathan Celli

**Affiliations:** 1 Department of Physics, University of Massachusetts at Boston, Boston, MA 02125, USA; ljubica.petrovic001@umb.edu; 2 Medical Physics Program, Department of Physics and Applied Physics, University of Massachusetts Lowell, Lowell, MA 02125, USA; moumano1@gmail.com; 3 ZEISS Group, Carl Zeiss Meditec, Inc., Dublin, CA 94568, USA; justin.hanlon@zeiss.com (J.H.); mark.arnoldussen@zeiss.com (M.A.); igor.koruga@zeiss.com (I.K.); 4 Dana-Farber/Harvard Cancer Center, Boston, MA 02215, USA; syasmin-karim@bwh.harvard.edu; 5 Department of Radiation Oncology, Johns Hopkins University, Washington, DC 20016, USA

**Keywords:** nanoparticles, cancer, 3D tumor models, pharmacokinetics, radiosensitization

## Abstract

Gold nanoparticles (GNPs) have shown particular promise as radiosensitizing agents and as complementary drug delivery agents to improve therapeutic index in cancer treatment. Optimal implementation, however, depends critically on the localization of GNPs at the time of irradiation, which, in turn, depends on their size, shape, and chemical functionalization, as well as organism-level pharmacokinetics and interactions with the tumor microenvironment. Here, we use in vitro 3D cultures of A549 lung carcinoma cells, which recapitulate interaction with extracellular matrix (ECM) components, combined with quantitative fluorescence imaging to study how time-dependent localization of ultrasmall GNPs in tumors and ECM impacts the degree of damage enhancement to tumor cells. Confocal imaging of fluorescence-labeled GNPs in 3D culture reveals that nanoparticles are initially embedded in ECM and only gradually accumulate in cancer cells over multiple days. Furthermore, the timing of GNP redistribution from ECM to cellular compartments directly impacts efficacy, with major damage enhancement when irradiation is performed after GNPs have accumulated significantly in 3D tumor nodules. These results underscore the importance of the timing and scheduling in treatment planning to ensure optimal radiosensitization, as well as the necessity of studying these effects in model systems that recapitulate elements of tumor microenvironment interaction.

## 1. Introduction

In the last two decades, advances in the development of nanoparticle-based platforms in medicine have shown promise for a wide range of applications in cancer imaging and therapeutics [1]. While relatively few nanotechnology-based therapeutic agents have yet to be approved by the FDA for cancer treatment, others are undergoing clinical evaluation and increasing implementation of cancer nanomedicine is anticipated [2]. Gold nanostructures, in particular, have shown exciting promise for both imaging and therapy [3,4]. The promise of gold nanoparticles (GNPs) to enhance radiotherapy has been widely reported [5,6]. GNPs are biocompatible, relatively nontoxic, and have a high atomic number favorable for interaction with photons to provide imaging contrast, with the production of micrometer-range photo-/Auger electrons, which, if localized in the target tissue, can enhance damage to tumors cells improving therapeutic index [4].

In addition to photon-induced emission of photo-/Auger electrons, biological mechanisms are expected to significantly contribute to GNP radiosensitization [7]. Some studies highlighting the biological effects have shown that the presence of GNPs may lead to cellular redox imbalance and eventually oxidative stress as a consequence of GNPs interaction with the cell membrane protein disulfide isomerase [8]. Others have demonstrated the capability of nanomaterials to inhibit the thiol-reductase (TrxR) protein family during radiosensitization [9] and also induce immune-mediated effects [10]. In general, biological pathways for radiosensitization may include ROS production, oxidative stress, mitochondrial dysfunction, cell-cycle effects, and DNA repair inhibition. Other possible biological mechanisms such as autophagy and endoplasmic reticulum (ER) stress have also been proposed [11]. These mechanisms of radiosensitization may account for the discrepancy between theoretical damage or dose enhancement ratios (DER) calculated for GNP, e.g., based on Monte Carlo simulations, compared with experimental DERs [12].

However, the discrepancy may also be in part due to assumptions made in the localization of GNPs. Many studies have emphasized that the extent of GNP-mediated radiation enhancement depends critically on spatial localization at the time of radiation delivery. Such spatial localization depends on the GNP size, shape and chemical functionalization, organism-level pharmacokinetics, and interactions with the tumor microenvironment. In a previous study [13], we developed ultrasmall PEGylated GNPs optimized for applications in radiotherapy. The nanoplatform has a number of key features including surface coating of heterobifunctional PEG with amine, carboxyl, methoxy functional groups, which make this a versatile nanoplatform to conjugate various moieties such as fluorophores, peptides, and drugs. The platform is also optimized for longer circulation, higher tumor uptake, modulated clearance, and high radiotherapy enhancement due to the small size. We also showed that these ultrasmall nanoparticles are optimal for radiotherapy applications because they would allow for appropriate intratumoral localization or distribution to accommodate radiotherapy schedules including for short-distance radiotherapy [14]. Using a formulation of ultrasmall 2–3 nm GNP, confocal bioimaging with monolayer cancer cell cultures showed robust uptake of GNPs in cells. Irradiation experiments showed greater than 2.8-fold tumor cell kill enhancement. However, more studies are needed, including in three-dimensional (3D) tumor models, to generate data that can inform optimal treatment planning parameters; therefore, ultrasmall platforms are considered for clinical translation.

Here, we examine how uptake and localization of these ultrasmall GNPs reported in previous studies may impact therapeutic efficacy using a 3D cell culture model of human lung cancer. This model system includes extracellular matrix (ECM) cues responsible for more physiologically relevant multicellular architecture, a feature that has increasingly motivated the adoption of 3D tumor models in general as a bridge from traditional monolayer cultures to more resource-intensive animal models [15,16]. In the culture system used in this study, cancer cells form multicellular nodules on a matrix of growth factor reduced (GFR) Matrigel. The resulting spheroids contain both surface-exposed (well-oxygenated) and deeply buried (hypoxic) cells, resembling the in vivo microenvironment. Typically, lower doses of virtually any intervention are required to induce cell death in monolayer than in 3D or in vivo. This is due both to drug penetration effects and well-documented multicellular resistance (MCR) that appears as a result of cell–cell contacts in cells organized into 3D tissues [17]. These 3D cell cultures obviously lack tumor vasculature that is present in animal models. The other side of this tradeoff is the capability for easily interrogating longitudinal changes in nanoparticle distribution at the tissue’s cellular and subcellular levels. The present study seeks to leverage this capability of 3D culture to gain insight into the questions of where GNPs go, and when do they reach there, as determinants of the extent of radiation enhancement. While the size and surface charge of GNPs have been examined as determinants of GNP delivery [18,19], this study examines specifically how the presence of ECM alters the kinetics of uptake and retention. In particular, this study focuses on characterizing the time-dependent shift from the extracellular matrix to nodular localization in 3D tumor models.

While the methods and findings of the present study are likely applicable to a broad range of solid tumors, the focus on lung cancer is motivated by the high lethality of the disease combined with the high potential for improvement using such GNP platforms, which can be administered intravenously or intratumorally using smart radiotherapy biomaterials, or by inhalation [14,20,21,22,23]. Lung cancer is the leading cause of cancer death among males, accounting for 24% of total cancer deaths and the 19% five-year survival rate has shown relatively poor improvement, compared with other forms of cancer [24]. Moreover, for radiotherapy treatment, the normal tissue damage that results from respiratory motion contributes to limiting the dose delivered and motivates strategies to improve the therapeutic index. The results should help inform the development of GNP-aided radiotherapy and image-guided radiotherapy in particular, as well as image-guided drug delivery, where such GNPs optimized for radiotherapy can also be loaded with drugs.

## 2. Materials and Methods

**Figure 1:**
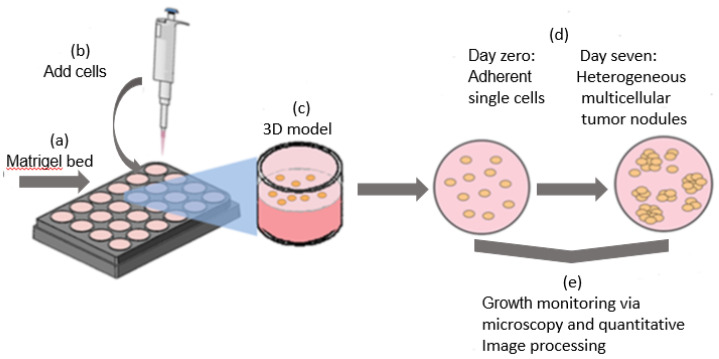
(**a**–**e**) The 3D cell culture model of A549 lung carcinoma cells: Cells were overlaid on ECM bed and allowed to grow for 7 days, leading to the formation of heterogeneous 3D tumor spheroids. Images were acquired longitudinally and analyzed to characterize growth behavior.

The 3D cell culture model. A549 human lung cancer cells were obtained from American Type Culture Collection (ATCC, Rockville, MD, USA) and maintained with RPMI-1640 medium with 10% fetal bovine serum, 1% penicillin–streptomycin solution, and 0.2% amphotericin B (HyClone Laboratories, Logan, Utah). Then, 3D cultures were plated in 24 black-walled glass-bottom plates (Greiner Bio-One, Solingen, Germany) or, for radiation experiments, in 35 mm glass-bottom culture dishes (MatTek Corporation, Ashland, MA, USA). ECM beds were prepared by transferring GFR Matrigel (Corning, Bedford, MA, USA) in the liquid phase at a temperature between 0 °C and 4 °C and performed on a chilled block, with previously chilled plates and pipette tips. Matrigel beds in plates or dishes were incubated at 37 °C for 20 min, to allow gelation prior to the addition of a single-cell suspension of cancer cells (Figure 1a). In the glass-bottom dishes, the ECM bed is formed only in the smaller inset area where the coverslip bottom sits. In multiwell plates, the ECM bed fully covered the bottom of each well. A single-cell suspension of 7500 cells per ml of A549 in a growth medium containing 2% Matrigel was added in each well (Figure 1b). It is worth noting that 3D cultures were typically grown in these conditions for seven days (Figure 1c,d) prior to administration of reagents and/or imaging, as described below.

GNP delivery. GNPs labeled with Alexa Fluor-594-Fab (Nanoprobes, Yaphank, NY, USA) were used for image-based monitoring of delivery and uptake. Fluorescent ultra-small GNPs about 1.4 nm in diameter were used as an indicator of particles accumulation period in the nodule interior. Fluorescent ultrasmall pegylated GNPs with a similar size range that have been well characterized and described in our previous studies [13] were also used for X-ray therapy enhancement studies. For fluorescent imaging, the concentration of fluorescently labeled nanoparticles was 0.0291 mg Au/mL. For treatment experiments, a concentration of 0.278 mg Au/mL was used, which amounted to 2.78 × 10^−3^ mg Au/well. Two methods were evaluated for the delivery of GNPs in 3D cell cultures. With a goal of approximating the in vivo scenario in which GNPs are delivered through the vasculature, the first approach was to directly add the GNPs in media overlaid on top of 3D cultures for 45 min, prior to being replaced with normal media. In this manner, nanoparticles have a finite time for uptake in an effort to model vascular circulation (as opposed to remaining in static supernatant for the full-time course of experiments). The second approach, modeling intratumorally delivery, was to directly inject the GNPs below the surface of the ECM bed at one injection site in the well center using a narrow gel loading pipette tip.

Radiation therapy. A custom research Petri dish irradiator, based on the IRay stereotactic radiotherapy system (Carl Zeiss Meditec, Dublin, CA, USA), was used as the X-ray delivery platform to irradiate cultured cells. In all experiments, X-ray delivery was performed at 100 kilovolts peak (kVp), generating X-rays with energies up to 100 keV with a spectral peak at 35 keV. The irradiator produces a circular beam that can accommodate a Petri dish of up to 60 mm in diameter. The beam produces a flat dose profile over the area such that the entire Petri dish is evenly irradiated, and by design, no part of the Petri dish is located in the penumbra. The system was calibrated to take attenuation and scatter effects into account.

Microscopy and image processing. The growth of 3D cultures was monitored via darkfield microscopy with a Zeiss Axio Observer Z1 inverted stage microscope.

Darkfield image data were processed in MATLAB to generate size distributions in terms of 2D area projection and an ellipsoid estimation of volume (*V*), as reported in previous studies using the same model [25,26], where a and b are the semi-major and semi-minor axes of the nodule, respectively:

$$V=\frac{4}{3}\pi ab^{2}.$$

To quantify uptake and localization of labeled GNPs in 3D cell culture, fluorescence and transmitted light images were acquired longitudinally by Zeiss Axio Observer Z1 and/or Zeiss LSM 880 confocal fluorescence microscope. The fluorescent signal in the multicellular nodules and surrounding ECM were quantified by segmentation and analysis of mean fluorescence intensity in the foreground (3D nodules) and background (ECM pixels, using the negative binary mask of the foreground). Image processing was performed in MATLAB using routines for quantification of fluorescence intensity in 3D cell cultures adapted from code described in previous reports [25,27].

GNP extraction and optical absorption measurements. For these measurements, 3D cultures were incubated with dispase, which enzymatically digests Matrigel. The cells were then separated by centrifugation, and the intensity of fluorophore absorption from lysates was measured in a BioTek Epoch multiwell plate reader.

## 3. Results

**Figure 2:**
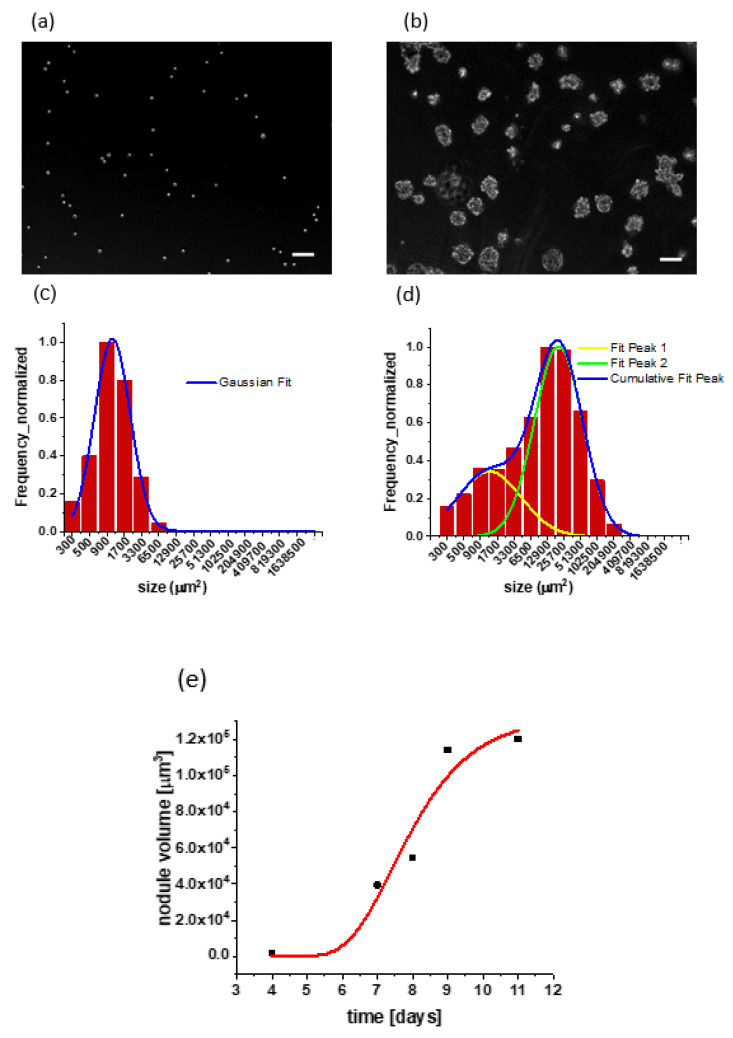
Longitudinal monitoring of 3D culture growth: (**a**) darkfield images show adherent single cells clearly visible at day zero. Scale bar is equal to 100µm; (**b**) heterogeneous multicellular tumor nodules are formed at day seven. Scale bar is equal to 100µm; (**c**) the first visible signs of heterogeneity appear on day four. Although one Gaussian fit is applied, the first two bars represent the beginning of another peak formation. The size of cancer nodules given in square micrometers corresponds to the area of a 2D projection (from 2D image data) of a 3D tumor nodule; (**d**) bimodal size distribution of 3D nodules at day seven in which the first peak corresponds to a smaller population with a mean size of about 1300 µm^2^, and the second peak includes a larger population with a mean size of 19,300 µm^2^; (**e**) growth kinetics plot (Gompertzian fit): average size of A549 3D nodule over time. The 3D nodule grew from day zero to day nine, when it reached a plateau due to stagnation in further growth.

The characterization of the 3D cell culture model. Growth behavior of in vitro 3D cultures was initially characterized by analysis of longitudinal darkfield microscopy images to establish 3D spheroid size distribution as relevant to nanoparticle uptake subsequently examined. Analysis of size distribution with respect to culture duration showed the emergence of a bimodal lognormal size distribution (Figure 2).

As reported previously for other cancer cells, cells overlaid on a GFR Matrigel bed formed heterogeneous 3D clusters through proliferation as well as comigration and aggregation [26]. At day zero (shortly after initial plating) the size distribution had a single Gaussian peak representing mostly single cells, as expected (Figure 2a). Analysis of images contained mostly single cells with typical rounded morphology observed in overlays on a soft hydrogel substrate and showed A549 cell diameter to be 19.3 µm. Over time, cells in 3D culture continued to form larger nodules through a combination of proliferation and aggregation events, as indicated by the shifting center position of the second peak in Figure 2d, though a smaller population of single cells and small nodules, represented by the first peak in the same figure, were still present. Proceeding with an analysis of the growth kinetics of the second (larger volume) peak, we found that this more aggressively growing population showed reasonable agreement with a Gompertzian growth model [28].

$$\;N=N_{0}e^{A(1-e^{-\alpha t)}/\alpha }\;$$

Series expansion at small times gives exponential growth behavior as follows:

$$\frac{N}{N_{0}}\approx e^{At}=2^{t/\tau }\;$$

where τ is doubling time. Using a Gompertzian curve coefficient ($A=0.79872\;{\mathrm{day}}^{-1}$) (Figure 2e) obtained via nonlinear least squares fit, the A549 3D culture exponential phase doubling time is calculated as

$$\tau =\frac{\ln2}{A}=20.83\;\mathrm{hours}.$$

Although this estimated volumetric doubling of 3D cultures contributed to both cell proliferation and nodular aggregation events, it was nevertheless reasonably close to previously reported 22 h [29]. The observation of a Gompterzian plateau at relatively small sizes is likely driven partly by the inherent incapability of these homocellular 3D cultures to develop new vasculature to supply oxygen and nutrients to the interior space of growing multicellular nodules.

**Figure 3:**
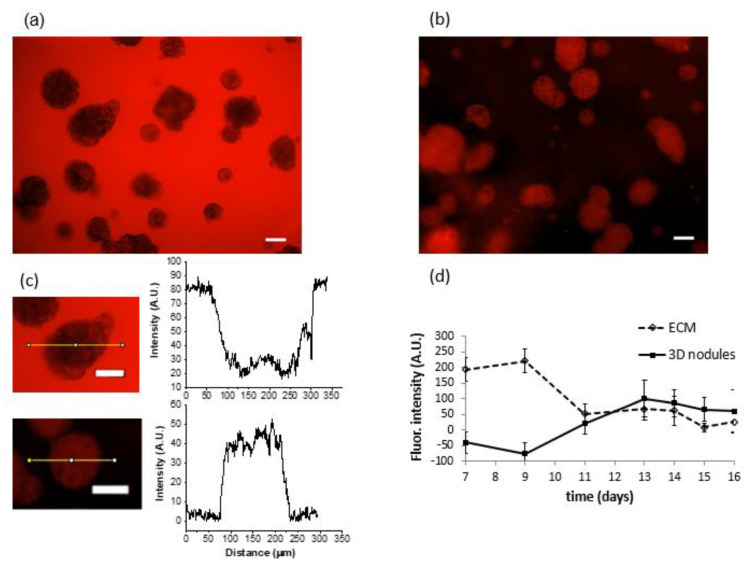
Longitudinal monitoring of fluorescence intensity from fluorophore-labeled GNPs: (**a**) after 45 min of cancer nodule exposure to GNPs, Matrigel appeared red due to GNP localization within the ECM. Due to a strong interaction of Matrigel and GNPs, particles were incapable of migrating immediately to cancer nodules and are retained in the matrix. Scale bar is equal to 100 µm; (**b**) a 3D nodule of A549 lung cancer at day 13, which was 6 days after exposure to GNPs. Most of the GNPs moved from the Matrigel to cancer nodules, which turned red due to fluorophore presence, while Matrigel appeared black. Scale bar is equal to 100 µm; (**c**) the intensity drop between Matrigel and a nodule at day 1, as well as the intensity jump at day 6, are also confirmed using ImageJ software. Scale bar is equal to 100 µm; (**d**) fluorophore intensity in the ECM (dashed line) and in the nodules (solid line) with standard error (SE), *n* = 3. Light emitted by fluorophore accumulation in Matrigel decreased over time unlike light intensity emitted by fluorophore in cancer cells. Light intensity emitted by tumor nodules constantly increased and reached the maximum at day 13, which was 6 days after the addition of GNPs. In order to obtain the appropriate intensity, a control group was used to determine the background, which was subtracted from each individual image.

Time-dependent GNP accumulation in ECM and tumor spheroids. Alexa Fluor-594-labeled GNPs were added to the culture medium on day seven. In an effort to model vascular delivery, in which particles would circulate through tissue rather than residing in static media solution, the nanoparticle-containing media was removed after 45 min of exposure, and cultures were returned to fresh media prior to imaging. Fluorescence signal from the Alexa Fluor-594-labeled particles was monitored daily, keeping the imaging parameters, including exposure time, zoom level, and objective-to-target distance the same for each imaging session. Images were taken from day 7, when GNPs were added (Figure 3a), until day 16. The maximum intensity of light emitted by the nodule was reached on day 13, which was 6 days after the addition of GNPs (Figure 3b).

**Figure 4:**
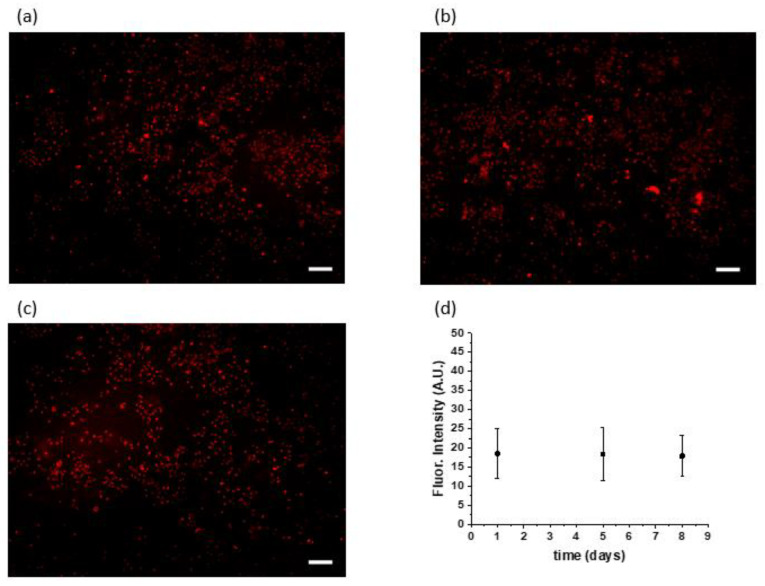
Monolayer cultures of A549 cancer cell line with GNPs inside at (**a**) day one, (**b**) day five, and (**c**) day eight. No significant difference was observed in GNP accumulation in cells over time, given that most GNPs are absorbed during the first 24 h; (**d**) *n* = 3. (**a**–**c**) Scale bar is equal to 200 µm.

The intensity of the fluorescence signal was obtained by processing images in MATLAB. A no-treatment group was used to determine the image background, which was subtracted from the intensity of all single images. Fluorescent images of the extracellular matrix (ECM) and nodules monitored daily gave a clear picture of the declining intensity in ECM and increasing intensity in nodules (Figure 3c). In other words, GNPs initially stuck to the matrix and then were gradually taken up by 3D tumor nodules. On day 13, that is, 6 days after the initial delivery of GNPs, the intensity of emitted light reached a plateau (Figure 3d, day 13–16), suggesting that maximal tumor delivery was finally achieved by this time. To further examine the in vitro model of GNP delivery, experiments were also performed in which GNPs were injected directly into ECM and compared with the addition of overlaid media covering the 3D culture. The retention time of GNPs was essentially the same in both cases. Confocal images of GNP accumulation provide additional insight into the time-dependent transport of GNPs from ECM to the nodule periphery, and then to intramodular and intracellular spaces. Importantly, a completely different situation was encountered in monolayer (2D) cultures of the same cancer cell line. A549 monolayers were exposed to GNPs for one day before washing and imaging (day one), and they were exposed to GNPs for five and eight days prior to washing and imaging (day five and day eight). It was found that GNPs delivered rapidly with no continued uptake at days five through eight (Figure 4). This observation is in stark contrast to the ECM-containing 3D cultures in which we observed a long and slow period of redistribution from extracellular to intracellular spaces.

The longer retention time of GNPs in Matrigel relative to media. The transport of GNPs delivered in culture medium (which is overlaid on top of ECM) is presumably determined by a combination of sedimentation and diffusion. Based on the small size of GNPs used in this study, we made the initial approximation that transport would be dominated primarily by diffusion, rather than sedimentation [30].

**Figure 5:**
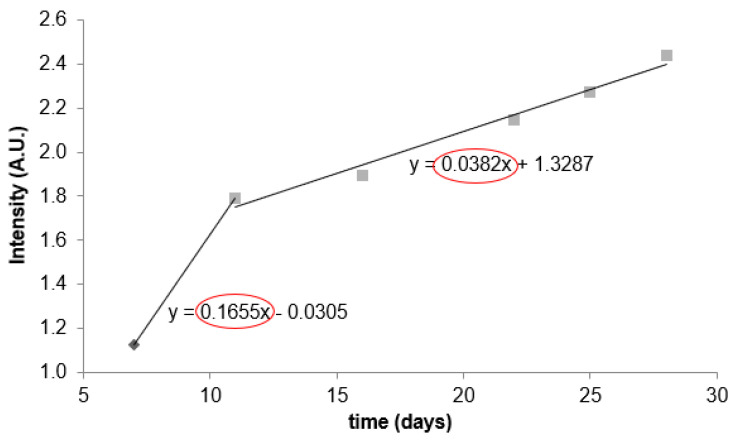
Intensity curve representing the ratio of the average nodule and Matrigel intensity over 28 days gives a broader picture of GNP behavior in a 3D model. GNP uptake by nodules was most rapid up to day 11 of nodule growth (during the first 4 days after the addition of labeled nanoparticles). After this period, uptake slowed down due to lower GNP concentration in Matrigel, confirmed by a slope 2 (11–28 days) value (0.0382), which is more than four times less than slope 1 (7–11 days) value (0.1655).

Longitudinal monitoring of GNP uptake. In order to evaluate longer-term uptake kinetics, cultures were monitored daily up to day 28. An intensity curve was obtained by dividing the average fluorescence intensity of GNPs localized in 3D nodules by the intensity from ECM (the ratio of image intensity multiplied by binary image mask and image intensity multiplied by the negative binary mask) (Figure 5).

From inspection of the intensity versus time data, it became evident that there were different rates of uptake: it was initially more rapid (approximately days 7–11) and then slower at later times (days 11–28). Treating each time window roughly linearly, the rate for days 7–11 was more than four times greater than that for days 11–28. Although GNPs were apparently embedded in ECM for extremely long durations (nearly a month), there was a window of relatively higher uptake in the first several days following administration, which makes sense for a diffusion-driven process.

**Figure 6:**
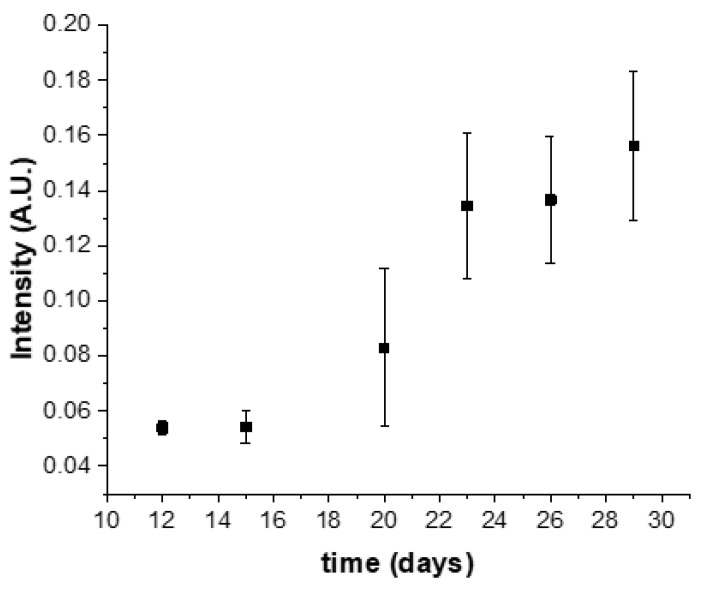
The intensity of light emitted by fluorescently labeled nanoparticles in A549 3D nodules over time with bars indicating standard error, *n* = 3. Cancer cells were periodically extracted from Matrigel by enzymatic matrix digestion and centrifugation, and the intensity of fluorophore absorption was measured in a plate reader. This absorption increased over time due to the time-dependent accumulation of GNPs in cancer cells.

Similar results were obtained by optical absorption measurements of GNPs via terminal measurements in cell lysates obtained at specific time points. In this case, replicate culture groups were harvested at the indicated time points by enzymatic digestion of ECM and centrifugation to separate cells, and cell lysates containing GNPs were transferred to a fresh multiwell plate and read by a plate reader (Figure 6). Although noisier than in situ imaging-based measurements shown above, the trend in uptake over time was in general agreement.

Time-dependent therapy enhancement. Based on image-based GNP uptake data, we hypothesized that radiation enhancement should be greater if radiation is delivered at around the cross-over point in matrix/nodular accumulation observed in imaging studies (Figure 3) rather than at an early time point within the first 24 h (which would be a more traditional choice for in vitro experiments). To test this, we delivered 2 Gy X-ray radiation to 3D cultures, which were staggered to receive GNP delivery one or four days prior to irradiation. Radiation response was assessed by clonogenic survival of cells from 3D cultures that were harvested and replated in 35 mm dishes to assess capability for colony formation of single cells after radiation damage [31].

**Figure 7:**
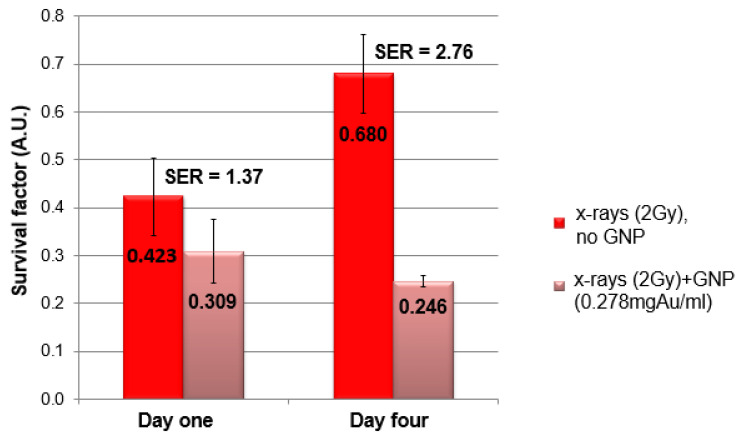
Radiation treatment response wo/w GNPs in a 3D model. Treatment enhancement depended on localization of GNPs. Bars with SE (*n* = 3) represent the post-radiation clonogenic survival factors wo/w GNPs after one day and four days of accumulation. The presence of GNPs enhanced radiation effectiveness, but a four-day accumulation period showed doubled SER, compared with one day (ml = mL).

Clonogenic assays (Figure 7) show that the degree of enhancement was indeed greater after a longer post-GNP delivery period. Here, the sensitivity enhancement ratio (SER), which is defined as the ratio of survival factor upon radiation without and with GNP, was 2.76 (0.680/0.246) after a four-day accumulation period, compared with 1.37 (0.423/0.309) upon one-day accumulation. SER more than doubled after allowing several days for redistribution of GNPs from the extracellular matrix into tumor nodules. Differing radiation response at each time point in the absence of GNPs was expected, as cultures became larger, more hypoxic, and radiation-resistant.

This is consistent with the expectation that a cascade of low-energy, short-range secondary particles are produced, which should enhance treatment response due to the dense deposition of their energy in the immediate vicinity of GNPs [32]. It is worth noting, however, that there can be a difference between survival factors in radiation-treated cultures across time points, which is related to the age of the cultures. In this study, 3D cultures were plated simultaneously so that cultures receiving radiation at any earlier time point were smaller (see growth analysis data above), with smaller internal hypoxic and treatment-resistant cores.

## 4. Discussion

Overall, the results showed that the time-dependent distribution of GNPs in tumors and ECM also affects damage enhancement or radiosensitization, as SER was doubled after a four-day accumulation period, compared with one day. This highlights the need for image-guided delivery of GNPs to ensure optimal radiosensitization. Given that GNPs provide imaging contrast, tumors can be imaged over time to determine optimal time points for radiotherapy.

As mentioned above, a custom research irradiator was used at 100 kVp, which may be most relevant for short-distance radiotherapy (brachytherapy) energies. The damage enhancement is expected to differ for higher energy external beam radiotherapy, in which case photoelectric interaction is expected to be less. In addition, when GNPs are used for other applications, the required accumulation or nanoparticle delivery period may change depending on other parameters such as functionalization.

This study could also be relevant to efforts using GNP-enhanced radiation to target components of the tumor microenvironment. For example, the concept of targeted depletion of tumor stroma has emerged as a therapeutic paradigm in the treatment of pancreatic cancer, a particularly lethal disease that is known for profound desmoplastic reaction and dense fibrotic stroma that inhibits drug delivery [33]. It is interesting that, in this study, we found that GNPs, without any deliberate functionalization to target stromal components, were deposited extensively in ECM. Although the present study emphasized delivery to tumor cells, one could also imagine looking at the inverse scenario—leveraging the short-term deposition of GNPs into stroma in order to intentionally increase radiation delivered to stroma prior to chemotherapy. Additionally, along these lines, targeting tumor vasculature is a strategy that has been extensively explored to cut off the flow of oxygen, nutrients, and routes of dissemination from tumors. In the same vein, in vivo imaging of vascular retention and extravasation can be used to inform vasculature-targeted GNP radiosensitization for intransigent or nonresectable tumors that exhibit poor response to other therapies [20].

The results reported here could also be directly applicable to the radiotherapy approach, in which smart radiotherapy biomaterials (SRBs) loaded with GNPs can release GNPs locally into the tumor tissue [4,21,22]. This approach is facilitated by the use of biodegradable polymers as part of the SRBs to enable GNP release in situ. Inhalation delivery of nanoparticles to lung tumors has also been considered [23,34]. Both scenarios require detailed knowledge of GNP localization and distribution within the tumor microenvironment at a particular time, as investigated in the present study.

Obviously, the use of 3D cell cultures, as carried out in the current study, does not completely model the tumor microenvironment. However, the resulting data provide additional insights relevant to the development of the ultrasmall nanoparticles optimized for radiotherapy applications and should inform animal studies, as such platforms are considered for clinical translation.

## 5. Conclusions

GNP-mediated radiosensitization is strongly dependent on the localization of nanoparticles at the time of radiotherapy and should be considered seriously in nanoparticle-aided radiotherapy. Here, we showed that GNPs were concentrated primarily in the surrounding ECM for surprisingly long durations and that significant nodular accumulation occurred several days after administration. This result was found using an in vitro 3D cell culture model that restored biologically relevant nodular architecture and ECM interaction, which is absent in previously conducted monolayer culture studies and enables feasibility of cellular-level monitoring that would be difficult to achieve in vivo. Overall, the results enforce that timing of radiotherapy following administration of ultrasmall gold nanoparticles is a significant factor in determining treatment outcomes for nanoparticle-aided radiotherapy and needs to be taken into account in treatment planning. At the same time, these results could have implications for strategies that seek to target stromal components, in which case X-ray delivery to GNPs at the time of maximal stromal accumulation may be desirable in strategies for stromal depletion to complement drug delivery.

## Data Availability

Data supporting reported results beyond what is reported in this manuscript are available upon reasonable request from the corresponding authors.
